# Concordance in parent and offspring cortico-basal ganglia white matter connectivity varies by parental history of major depressive disorder and early parental care

**DOI:** 10.1093/scan/nsaa118

**Published:** 2020-08-25

**Authors:** Eyal Abraham, Jonathan Posner, Priya J Wickramaratne, Natalie Aw, Milenna T van Dijk, Jiook Cha, Myrna M Weissman, Ardesheer Talati

**Affiliations:** Department of Psychiatry, Columbia University Vagelos College of Physicians and Surgeons, New York, NY, USA; Divisions of Translational Epidemiology, New York State Psychiatric Institute, New York, NY, USA; Department of Psychiatry, Columbia University Vagelos College of Physicians and Surgeons, New York, NY, USA; Child Psychiatry, New York State Psychiatric Institute, New York, NY, USA; Department of Psychiatry, Columbia University Vagelos College of Physicians and Surgeons, New York, NY, USA; Divisions of Translational Epidemiology, New York State Psychiatric Institute, New York, NY, USA; Biostatistics, Mailman School of Public Health, Columbia University, New York, NY, USA; Department of Psychiatry, Columbia University Vagelos College of Physicians and Surgeons, New York, NY, USA; Child Psychiatry, New York State Psychiatric Institute, New York, NY, USA; Department of Psychiatry, Columbia University Vagelos College of Physicians and Surgeons, New York, NY, USA; Divisions of Translational Epidemiology, New York State Psychiatric Institute, New York, NY, USA; Department of Psychiatry, Columbia University Vagelos College of Physicians and Surgeons, New York, NY, USA; Child Psychiatry, New York State Psychiatric Institute, New York, NY, USA; Department of Psychiatry, Columbia University Vagelos College of Physicians and Surgeons, New York, NY, USA; Divisions of Translational Epidemiology, New York State Psychiatric Institute, New York, NY, USA; Departments of Epidemiology, New York, NY, USA; Department of Psychiatry, Columbia University Vagelos College of Physicians and Surgeons, New York, NY, USA; Divisions of Translational Epidemiology, New York State Psychiatric Institute, New York, NY, USA

**Keywords:** concordance, parent–offspring bonding, major depression, DTI, social cognition

## Abstract

Social behavior is transmitted cross-generationally through coordinated behavior within attachment bonds. Parental depression and poor parental care are major risks for disruptions of such coordination and are associated with offspring’s psychopathology and interpersonal dysfunction. Given the key role of the cortico-basal ganglia (CBG) circuits in social communication, we examined similarities (concordance) of parent–offspring CBG white matter (WM) connections and how parental history of major depressive disorder (MDD) and early parental care moderate these similarities. We imaged 44 parent–offspring dyads and investigated WM connections between basal-ganglia seeds and selected regions in temporal cortex using diffusion tensor imaging (DTI) tractography. We found significant concordance in parent–offspring strength of CBG WM connections, moderated by parental lifetime-MDD and care. The results showed diminished neural concordance among dyads with a depressed parent and that better parental care predicted greater concordance, which also provided a protective buffer against attenuated concordance among dyads with a depressed parent. Our findings provide the first neurobiological evidence of concordance between parents-offspring in WM tracts and that concordance is diminished in families where parents have lifetime-MDD. This disruption may be a risk factor for intergenerational transmission of psychopathology. Findings emphasize the long-term role of early caregiving in shaping the neural concordance among at-risk and affected dyads.

## Introduction

Humans are social by nature and biologically prepared to connect with others to promote their survival (Bowlby, 1969; Hardy, 2009). The parent–offspring bond, the first and most significant social relationship throughout life, is the context of offspring physical, social-emotional and cognitive growth (Sroufe, 1988). Therefore, offspring who experience chronic familial stressors, such as parental depression and/or poor parental care, are vulnerable to a myriad of adverse outcomes throughout life, including depression and other psychiatric illnesses, as well as social difficulties such as socio-cognitive deficits and behavioral problems (Cummings *et al.*, 2005; Weissman *et al.*, 2016; Weissman, 2020).

As mammals, our brain develops through the process of ‘biobehavioral synchrony’, the on-going exchanges of behavioral, hormonal and physiological signals between parent and offspring during social contact (Feldman, 2012). In humans, concordance in parent–offspring physiological processes is based not only on proximity and touch, as seen in other mammals (Curley and Champagne, 2016), but also on shared empathic experiences, social understanding and perspective-taking, and has been suggested as a potential mechanism underlying human social bonding (Creaven *et al.*, 2014; Ebisch *et al.*, 2012; Feldman *et al.*, 2010, 2011; Mayo and Gordon, 2020; van Bakel and Riksen-Walraven, 2008).

Parent–offspring concordance of social cues and physiological processes (e.g. hormones, heart rhythms, respiratory sinus arrhythmia) during childhood and adolescence provides critical inputs to offspring optimal neurobehavioral development. Parent–offspring concordance supports offspring’s participation in social life, including development of self-regulation, empathy and symbolic skills in early childhood, and pro-sociality and social cognition later in life, which in turn support the ability to form and maintain social relationships and to parent the next generation (Abraham *et al.*, 2014, 2016; Feldman, 2012, 2017). Studies in animals (Perkeybile and Bales, 2017) and humans (Abraham *et al.*, 2017; Feldman, 2017) have shown that intergenerational transmission of social behavior is modulated by early parental care and regulated by the cortico-basal ganglia (CBG) circuits during moments of interpersonal synchrony (Schirmer *et al.*, 2016).

The human basal ganglia, including the caudate, putamen, nucleus accumbens (NAcc) and pallidum, is a central node of the subcortical dopaminergic motivation/reward circuitry that supports affective representation of others and multiple social goals by integrating reward-related learning, motivation, motor control and habit formation (Delgado, 2007; Báez-Mendoza and Schultz, 2013). Its connections with cortical regions involved in empathy, simulation and theory-of-mind (ToM), are translated into higher-order social behavior by providing incentive for long-term social goals (Shamay-Tsoory, 2011; Gordon *et al.*, 2013; Van Den Bos *et al.*, 2015), and by using reward outcomes to guide social cognition and form stable attachments, including parent–offspring bonding, pair-bonding and friendships (Rilling and Young, 2014; Tops *et al.*, 2014). Two systems in the mesocorticolimbic pathway—dopamine (DA) and oxytocin (OT) form tighter crosstalk during periods of bond formation, including the parent–offspring bonding, to reorganize neurobiological systems implicated in emotion, reward and motivation processes around social experiences (Feldman, 2017; Ulmer-Yaniv *et al.*, 2016). The CBG circuit holds particular importance in the context of parental depression since its development is sensitive to early experiences within child-rearing contexts, as well as its central role in underpinning maternal and paternal care, and in co-wiring of parent’s and offspring’s brains and behavior into a synchronous unit that supports the offspring’s brain development (Skelin *et al.*, 2015; Nusslock and Miller, 2016; Qu *et al.*, 2016; Feldman, 2017). Also, aberrant functionality and connectivity of CBG circuits along with deficits in multiple social cognitive domains were found to be associated with depression (Marchand *et al.*, 2012; Ma *et al.*, 2012; Gabbay *et al.*, 2013).

While parent–offspring concordance has been demonstrated on behavioral, arousal and hormonal levels, much less is known about how two brains coordinate in the context of social bonding, and how neural concordance varies by risk factors such as parental depression and poor parental care. Recently, neuroimaging research in healthy population has pinpointed dyadic neural concordance in the basal ganglia (Stephens *et al.*, 2010), and mainly in temporal cortical structures implicated in social communication, including the fusiform gyrus (FG)/inferior temporal gyrus (ITG), superior temporal sulcus (STS) and temporoparietal junction (TPJ). This neural concordance was associated with degree of social connectedness and the quality of relationship, such as cooperation and its success, and with greater shared intentionality between partners (e.g. Atzil *et al.*, 2012; Cui *et al.*, 2012; Goldstein *et al.*, 2018; Hasson, 2004; Kinreich *et al.*, 2017; Levy *et al.*, 2017; Meyer *et al.*, 2019; Miller *et al.*, 2019; Mu *et al.*, 2016; Stephens *et al.*, 2010; Tang *et al.*, 2016). Only two studies measured concordance in neural structure and activity in at-risk sample of dyads with a depressed mother and found that depressed mothers’ cortical thinning in middle temporal gyrus (MTG; Foland-Ross *et al.*, 2016) and activation of the putamen (Colich *et al.*, 2017) predicted their never-depressed daughters’ cortical thinning and activation in the same areas, respectively. No studies which we are aware of have examined dyadic concordance in parents and offspring CBG white matter (WM) connectivity and how such concordance varies by parental major depressive disorder (MDD) status and early parental care.

**Fig. 1.**
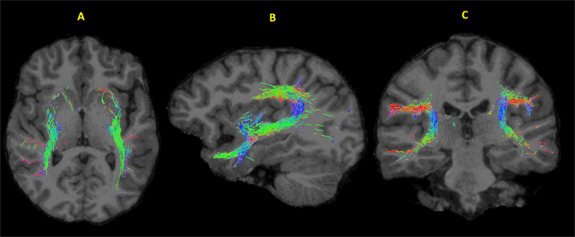
Cortico-basal ganglia white matter connectivity.

Given the role of aberrant social-cognitive processing in the profound and pervasive social impairments displayed by individuals with MDD (Tse and Bond, 2004), we examined dyadic concordance in parents and offspring CBG WM connectivity in 44 dyads from a sample of three generations of families followed prospectively up to 40 years (Weissman *et al.*, 2016), comprising parents (second generation) with and without a lifetime history of major depression disorder (MDD) and their depressed and never-depressed offspring (third generation). We used diffusion tensor imaging (DTI) tractography to examine parent–offspring concordance of the integrity of WM tracts, which connect the basal ganglia nuclei with six temporal regions, all of which implicate in mental representation of others, social communication and parent–offspring bonding. Regions were selected based on recent social neuroscience literature on social cognition and brain coupling (for review see: Van Overwalle, 2009; Shamay-Tsoory, 2011; Adolfi *et al.*, 2017; Long *et al.*, 2020) (Figure 1).

Illustration of the white matter fiber tracks connecting the basal ganglia with the temporal regions of interest. A, axial; B, coronal; C, sagittal views.

To assess concordance between parents and offspring, we examined the similarities (as indicated by positive correlations) of parent and offspring CBG connections. We hypothesized that CBG connection strength would correlate in parent and offspring above and beyond parent MDD status (Hypothesis 1). Second, we investigated whether and how parent-level risk factors for psychopathology and social cognition impairments—parent lifetime-MDD and low parental care—would moderate these associations. Based on previous work demonstrating that parental depression is associated with attenuated dyadic behavioral (Field *et al.*, 1990; Murray and Cooper, 1996; Tronick and Reck, 2009; Granat *et al.*, 2017), hormonal (Merwin *et al.*, 2017), and autonomic physiology (Woody *et al.*, 2016) coordination, we hypothesized that parental lifetime-MDD would be associated with reduced or diminished parent–offspring neural concordance (Hypothesis 2). We assessed early parental care using the Parental Bonding Instrument (PBI), which is an instrument to measure parent–child bonding during the first 16 years of life (Parker *et al.*, 1979) on the parental Care dimension (sensitive and responsive parenting). Given evidence that individual differences in quality of early parent–offspring relationship play an important role in physiological concordance (Atzil *et al.*, 2012; Smith *et al.*, 2016; Suveg *et al.*, 2019), we hypothesized that higher levels of parental Care during childhood, as reported by offspring, would be associated with a greater parent–offspring concordance of CBG WM connections (Hypothesis 3). Finally, since social attachments act as a protective buffer against many negative consequences of stress and adversity, including parental depression (Hagan *et al.*, 2011; McNeal *et al.*, 2017), and not all depressed parents necessarily display poor parental care (Flykt *et al.*, 2010), we sought to determine if higher levels of parental Care can protect the attenuated dyadic concordance from the effects of parental lifetime-MDD (Hypothesis 4).

## Materials and methods

### Participants

The study began in 1982, with recruitment of adults (probands, generation one, G1) with moderate to severe MDD seeking treatment at outpatient facilities; and a comparison group of adults from the same community with no lifetime psychopathology, as determined by several interviews. The G1 probands, as well as their children (G2) followed over up to six assessment waves, corresponding to year 0 (Baseline), 2, 10, 20, 25, and 30. G3 (grandchildren) aged into the study starting time 10 or 20. G2/G3 offspring of the probands with MDD constituted the high-risk for depression group, and those of probands without psychopathology, the low risk group. Complete details on study design, sample selection, and assessments are reported elsewhere (Weissman *et al.*, 2016). This study is based on year 30 of the study, at which time the magnetic resonance imaging (MRI)/DTI assessment was completed. The Institutional Review Board of the New York State Psychiatric Institute (NYSPI) approved the study procedures. Adult participants provided informed consent; minors provided informed assent, and a parent/guardian provided consent. A total of 66 participants, consisting of 27 (16 mothers) G2 parents aged 39–59 years and their 39 (19 daughters) biological G3 offspring aged 10 to 31 years, were included. These G2 and G3 participants were distributed into 44 biological parent–offspring dyads, consisting of 24 dyads with parents diagnosed with Lifetime-MDD, and 20 dyads with never-MDD parents (Table 1). All participants were Caucasian, and G1 participants were all drawn from the same community. Exclusion criteria consisted of psychotic symptoms, pregnancy and MRI contraindications.

**Table 1.** Characteristics of study parents and offspring

	Total sample	MDD parent—Never-MDD offspring dyads (numbers, %)	MDD parent-MDD offspring dyads (numbers, %)	Never-MDD parent-MDD offspring dyads (numbers, %)	Never-MDD parent—Never-MDD offspring dyads (numbers, %)	Test statistic	*P*-value
**Number of dyads**	44	17 (38.6%)	7 (15.9%)	6 (13.6%)	14 (31.8%)		
**Number of participants:**	66	**Lifetime-MDD**		**Never-MDD**			
Second generation (G2)—parents	27	16 (59%)		11 (41%)			
Third generation (G3)—offspring	39	10 (25.6%)		29 (74.4%)			
**Age (years):**							
Second generation (G2)	Mean ± SE: 48.20 ± 0.68 Range: 38.9–59.6	47.1 ± 0.82	49.6 ± 2.3	47.8 ± 1.77	48.9 ± 1.37	F_(3,41)_ = 0.642	0.593
Third generation (G3)	Mean ± SE: 20.28 ± 0.71 Range: 10.7–31.4	19.04 ± 0.82	20.9 ± 1.975	21.9 ± 2.24	20.7 ± 1.53	F_(3,41)_ = 0.712	0.551
Parent (G2)-offspring (G3) age interval	Mean ± SE: 27.9 ± 0.65 Range: 19.20–38.30	28.30 ± 0.08	28.83 ± 0.45	25.82 ± 0.59	28.4 ± 0.06	F_(3,41)_ = 1.326	0.256
**Gender:**							
Second generation (G2) participants/dyads							
Male	Participants: 11 (40.7%) Dyads: 21 (47.7%)	9 (52.9%)	3 (42.9%)	5 (83.3%)	4 (28.6%)	*X* ^2^ = 5.36	0.147
Female	Participants: 16 (59.3%) Dyads: 23 (52.3%)	8 (47.1%)	4 (57.1%)	1 (16.7%)	10 (71.4%)		
Third generation (G3) Participants/dyads							
Male	Participants: 20 (51.3%) Dyads: 21 (47.7)	8 (47.1%)	2 (28.6%)	5 (83.3%)	6 (42.9%)	*X* ^2^ = 4.21	0.239
Female	Participants: 19 (48.7%) Dyads: 23 (52.3%)	9 (52.9%)	5 (71.4%)	1 (16.7%)	8 (57.1%)		

Values are mean ± SE unless specified.

### MRI and DTI data

Structural MRI (voxel = 1 m^3^, dimensions = 256 × 256 × 162) and diffusion MRI (voxel = 0.94 × 0.94 × 2.5 mm^3^, dimensions = 256 × 256 × 58, b = 1000 sm^-2^, direction = 15) scans were acquired on a GE 3T scanner. Cortical and subcortical parcellations of the structural MRI scans were performed with Freesurfer (http://freesurfer.net), and these parcellations were used for individualized seed and target regions for tractography, as described below.

To quantify the number of streamline counts, an index of structural connectivity, diffusion MRI scans were processed with MRtrix (https://www.mrtrix.org/; Tournier *et al.*, 2019). Briefly, with this approach, diffusion MRI scans are first denoised using random matrix theory allowing for a data-driven threshold for Principal Component Analysis denoising (Veraart *et al.*, 2016). This denoising method enhances quantitative and statistical interpretation (Veraart *et al.*, 2016). Denoised images then undergo eddy current and motion correction, brain extraction from three non-diffusion-weighted images (taking their median), and bias field correction using an N4 algorithm (N4ITK) in Advanced Normalization Tools (ANTs) (Andersson and Sotiropoulos, 2016; Tustison *et al.*, 2010). Fiber orientation distributions are then estimated from the preprocessed diffusion MRI scans and probabilistic tractography is performed based on second-order integration over fiber orientation distributions (iFOD2) with a target streamline count of 10 million (Tournier *et al.*, 2019). Tractograms are filtered using spherical-deconvolution informed filtering of tractograms (SIFT) with a streamline count target of 1 million. SIFT permits mapping of the streamline estimation to an individual’s diffusion MRI scan in addition to updating the streamline reconstruction to improve model fit. Finally, connectivity matrices were generated for each participant using the brain parcellation and segmentation obtained from the structural MRI scan from the same person. In this way, the structural connectome estimates reflect individualized connectomes constrained by the individual’s neuroanatomy. A prior macaque study suggests the validity of streamline counts as an indicator of fiber connection strength with the number of streamlines significantly correlating with tract-tracing strength in the macaque brain (van der Heuvel *et al.*, 2015).

The following steps were taken to address potential effects of head motion during MRI scanning. First, the diffusion weighted scans were manually inspected volume by volume by a trained technician prior to any processing. No volumes with motion or other imaging artifacts were identified during manual inspection, and thus, all volumes were used for analysis. Second, rotational and translational head motion parameters were calculated for each scan. We ran independent sample t-tests and no statistically significant differences average rotation and average translations were found between high *vs* low risk and lifetime-MDD *vs* no MDD and high *vs* low parental Care (*P* > 0.05; see Supplementary information Table S1). Head motion was minimal (average rotation < 0.01 mm and average translation < 0.6 mm).

Region of interest (ROI) included four basal-ganglia seed regions: the NAcc, caudate, putamen and pallidum, and six cortical regions within the temporal lobe. We chose to focus on regions within the temporal lobe due to their important role in social cognitive processing (Van Overwalle, 2009), and the fact that these regions support brain-to-brain neural synchrony (Dikker *et al.*, 2014; Jiang *et al.*, 2012; Liu *et al.*, 2017; Tang *et al.*, 2016). All six temporal regions, including the STS, MTG, ITG, FG, TPJ and temporal pole (TP) (Figure 1) were commonly found in ToM, empathy, brain coupling and social communication paradigms.

### The schedule for affective disorders and schizophrenia

At each wave, MDD was diagnosed using the Schedule for Affective Disorders and Schizophrenia-Lifetime Version; the adult version for participants over age 18 years (Mannuzza *et al.*, 1986) and the child version for participants 6 to 17 years of age modified for DSM-IV for children and adolescents (Kaufman *et al.*, 1997). Each family member was interviewed independently and blinded to the clinical status of other family members, by trained doctoral‐ and masters‐level mental health professionals (reliability, which was high, has been documented elsewhere (Weissman *et al.*, 2016). Final diagnoses were made by an experienced (Ph.D. or M.D. clinician) using the best estimate procedure (Leckman, 1982).

### Parent–offspring bonding

Parental Care was measured by the PBI, a widely used, validated and reliable measure of parent-child bonding (Parker *et al.*, 1979; Parker, 1990) at time 30. The PBI has survived many tests and years and remains an important clinical moderator of outcomes in intergenerational research nearly 45 years after it was introduced (Wilhelm *et al.*, 2005). Offspring (G3 = Grandchildren of G1, children of G2) were asked to complete the PBI on the specific parent (how the offspring experienced being parented by her/his parents). The PBI consists of 25 four-point items assessing the perception of parenting style/behaviors of each parent. Participants indicated how much their parents were like each statement on a 4-point scale (0 = Not at all to 3 = Always). The respondents answer the questions based on how they remember their parents during their first 16 years of life. Parents were evaluated along two dimensions, Care (sensitive and responsive parenting) and Overprotection (intrusive and excessive controlling parenting), with separate norms on each dimension for mothers and fathers. Parental Care subscale was used in the current study since research has shown that the parental Care dimension found to be more predictive for offspring depressive symptoms and diagnosis (moderate to large effect size) than the ‘Overprotection’ dimension (Alloy *et al.*, 2006). Still, after completing running the analyses based on our hypotheses, we examined the interactive effect of parental ‘Overprotection’ and parent’s strength of CBG WM connections on concordance, and consistent with the literature, there were no significant effect of overprotection. Although the responses rely on the subjects’ own recollections, the validity of the instrument has been supported by studies that show that subjects’ ratings correlate strongly with ratings of their parents themselves, siblings and impartial raters, including observational assessments of parental behavior (Parker, 1983; Steiger *et al.*, 1989). Age, sex and social class have minimal effect on scores. Also, by administering the PBI to depressed patients and repeating this when they remitted, it has been shown that the Care and Overprotection scores were stable over 20 years (Wilhelm *et al.*, 2005; Murphy *et al.*, 2010). In addition, to make sure the effect of PBI was not confounded by offspring age and sex in our sample, we used age and sex of the participants when they rated their relationships with their parents as covariates, and also we did not find any significant association between age and sex and rating of parental Care (r = 0.138, *P *= 0.371; t_(1,42)_ = − 0.126, *P *= 0.9, respectively).

## Analytic strategy

Parent–offspring concordance was defined as the standardized beta coefficient corresponding to the effect of parental WM connectivity on offspring WM connectivity (the number of fiber tracts at the level of streamlines) obtained from a generalized linear model. We used the generalized estimating equation (GEE) statistical approach (Liang and Zeger, 1986) in the framework of a generalized linear model to adjust for potential correlation of outcomes between siblings in the same family. These potential correlations among the outcomes were modeled as exchangeable. We controlled for familial (G1 depression status) risk status (High *vs* Low), offspring MDD status and parents’ and offspring’s age and sex (including parental MDD in Hypothesis 1). Because 48 connections (6 cortical regions × 4 basal ganglia regions × 2 hemispheres) were tested per hypothesis (Hypotheses 1–3), and 3 connections for Hypothesis 4, our four hypotheses yielded a total of 147 tests. In situations such as this, it is appropriate to control the false discovery rate (FDR) using the Benjamini-Hochberg (B-H) procedure (Benjamini and Hochberg, 1995), which is also the most common procedure in MRI studies. Under this procedure, the adjusted alpha level that yields an overall FDR of 5% is 0.01, so we report all *P*-values less than 0.01 as significant, under this adjustment. We used the PROC GENMOD procedure in SAS 9.4 (SAS Institute, NC, USA) to conduct these analyses.

## Results

### Intergenerational concordance in WM connectivity

We first sought to examine associations (concordance) in WM connectivity in CBG circuits between parents and their offspring (Hypothesis 1). We z-scored parameter estimates and conducted a series of GEE analyses and used familial (G1 depression status) risk status, parent and offspring MDD status, sex and age as covariates. Results of the significant associations are shown in Table 2 (for all tested associations, see Table S2), revealing several positive associations between parent’s and offspring’s strength of WM connections within the CBG circuits; between the NAcc and FG, ITG and TP, between the caudate and FG and STS, between the pallidum and TPJ/supramarginal gyrus (SMG) and TP, and between putamen and FG and TPJ, *P* < 0.05 FDR-corrected for multiple comparisons.

**Table 2.** White matter connections in CBG circuits showing significant associations* (concordance) between parents (with and without lifetime-MDD) and their offspring (with and without lifetime-MDD) and significant interaction effects* (a) between parent’s lifetime-MDD status × parent WM connectivity and (b) between parental Care × parent WM connectivity in predicting offspring WM connectivity

Basal ganglia region	CBG WM connections		L hemisphere			R hemisphere		
			Standardized ß (SE)	Z	*P*-value	Standardized ß (SE)	Z	*P*-value
NAcc—	Fusiform gyrus	Parent WM connectivity	0.38 (0.08)	4.35	<0.0001	-	-	-
		(a) Parental MDD × Parent WM connectivity	-	-	-	-	-	-
		(b) Parental Care × Parent WM connectivity	-	-	-	-	-	-
	ITG	Parent WM connectivity	0.34 (0.13)	2.59	0.001	-	-	-
		a	-	-	-	-	-	-
		b	-	-	-	-	-	-
	MTG	Parent WM connectivity	-	-	-	-	-	-
		a	0.42 (0.12)	3.26	0.001	-	-	-
		b	-	-	-	-	-	-
	Temporal pole	Parent WM connectivity	-	-	-	0.48 (0.12)	3.9	<0.0001
		a	-	-	-	-	-	-
		b	-	-	-	-	-	-
	STS	Parent WM connectivity	-	-	-	-	-	-
		a	-	-	-	-	-	-
		b	-	-	-	1.57 (0.53)	2.9	0.003
	TPJ/SMG	Parent WM connectivity	-	-	-	-	-	-
		a	-	-	-	-	-	-
		b	-	-	-	-	-	-
Caudate	Fusiform gyrus	Parent WM connectivity	0.39 (0.14)	2.75	0.001	-	-	-
		a	-	-	-	-	-	-
		b	0.45 (0.16)	2.83	0.004	-	-	-
	ITG	Parent WM connectivity	-	-	-	-	-	-
		a	-	-	-	-	-	-
		b	-	-	-	-	-	-
	MTG	Parent WM connectivity	-	-	-	-	-	-
		a	-	-	-	-	-	-
		b	-	-	-	-	-	-
	Temporal pole	Parent WM connectivity	-	-	-	-	-	-
		a	-	-	-	-	-	-
		b	1.03 (0.32)	3.17	0.001	-	-	-
	STS	Parent WM connectivity	0.24 (0.11)	2.59	0.01	-	-	-
		a	-	-	-	-	-	-
		b	-	-	-	-	-	-
	TPJ/SMG	Parent WM connectivity	-	-	-	-	-	-
		a	-	-	-	-	-	-
		b	0.86 (0.44)	1.93	0.005	-	-	-
Putamen	Fusiform gyrus	Parent WM connectivity	-	-	-	0.28 (0.09)	3.02	0.002
		a	0.42 (0.12)	3.48	0.0005	-	-	-
		b	-	-	-	-	-	-
	ITG	Parent WM connectivity	-	-	-	-	-	-
		a	-	-	-	-	-	-
		b	0.33 (0.08)	3.87	0.0001	-	-	-
	MTG	Parent WM connectivity	-	-	-	-	-	-
		a	-	-	-	-	-	-
		b	-	-	-	-	-	-
	Temporal pole	Parent WM connectivity	-	-	-	-	-	-
		a	-	-	-	-	-	-
		b	-	-	-	-	-	-
	STS	Parent WM connectivity	-	-	-	-	-	-
		a	-	-	-	-	-	-
		b	-	-	-	-	-	-
	TPJ/SMG	Parent WM connectivity	-	-	-	0.57 (0.09)	6.16	<0.0001
		a	-	-	-	0.25 (0.08)	3.01	0.002
		b	0.41 (0.08)	4.93	<0.0001	-	-	-
Pallidum	Fusiform gyrus	Parent WM connectivity	-	-	-	-	-	-
		a	-	-	-	-	-	-
		b				1.42 (0.50)	2.83	0.004
	ITG	Parent WM connectivity	-	-	-	-	-	-
		a	-	-	-	-	-	-
		b	-	-	-	-	-	-
	MTG	Parent WM connectivity	-	-	-	-	-	-
		a	-	-	-	0.30 (0.13)	2.23	0.002
		b	-	-	-	0.52 (0.23)	2.26	0.002
	Temporal pole	Parent WM connectivity	-	-	-	0.30 (0.07)	4.18	0.0002
		a	-	-	-	-	-	-
		b				0.38 (0.11)	3.34	0.0008
	STS	Parent WM connectivity	-	-	-	-	-	-
		a	-	-	-	0.33 (0.13)	2.55	0.001
		b	-	-	-	0.58 (0.17)	3.30	0.001
	TPJ/SMG	Parent WM connectivity	0.49 (0.13)	3.65	0.002	0.24 (0.09)	2.56	0.01
		a	-	-	-	0.56 (0.26)	2.12	0.003
		b	0.52 (0.09)	5.29	<0.0001	0.85 (0.27)	3.13	0.001

*Significant associations and interactions after *P* < 0.05 FDR correction (*P* ≤ 0.01); ‘-’ indicates a non-significant association and interactions after FDR correction. For full table with all tested association and interactions, see Supplementary Information, Table S2.

### Parental lifetime-MDD and parental Care as moderators

**Fig. 2.**
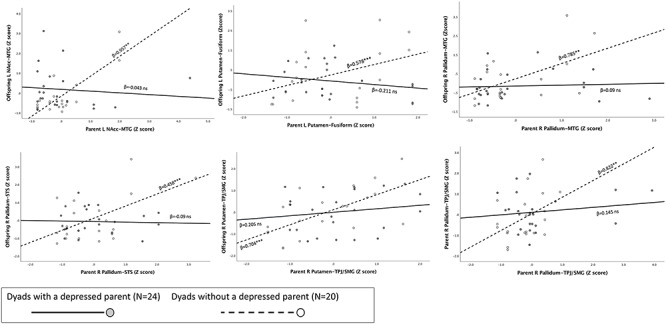
Graphical depiction of association between parent and offspring CBG WM connectivity by parent’s lifetime-MDD status, with stronger associations evident in dyads with a never-depressed parent. **P < 0.01; ***P < 0.001, ns, non-significance.

To test the moderating effects of parental history of depression (Hypothesis 2) and parental Care (Hypothesis 3) variables on parent–offspring concordance, we ran GEE analysis for each of the CBG connections. In each model, we entered parent lifetime-MDD status and parental Care as moderators of the effect of parental WM connectivity on offspring WM connectivity, and used familial (G1) risk status, offspring’s MDD status, parent and offspring sex and age as covariates. Significant findings (*P* < 0.05 FDR corrected for multiple comparisons) are presented in Table 2 (for all tested interactions, see Table S2). As can be seen, parent MDD interaction terms were significant in six CBG connections—L NAcc-MTG, L Putamen-FG, R putamen-TPJ, R pallidum-MTG, R pallidum-STS, and R pallidum-TPJ, indicating that dyads with a depressed parent and dyads without a depressed parent differed in the concordance between parent and offspring CBG connectivity. To illuminate the nature of the interactions, we plotted regression slopes of parent’s WM connectivity on the offspring’s WM connectivity for dyads with and without a lifetime-MDD parent (Figure 2). Simple slope analyses revealed that while among dyads with a never-depressed parent there were significant positive associations between parent’s and offspring’ strength of WM connections, there were no such associations among dyads with a depressed parent. As for parental Care, interaction terms were significant in 12 CBG connections; R NAcc-STS, L caudate-fusiform, L caudate-TP, L caudate-TPJ, L putamen-ITG, L putamen-TPJ, R pallidum-fusiform, R pallidum-MTG, R pallidum-TP, R pallidum-STS, and L/R pallidum-TPJ, indicating that the better the quality of early caregiving (e.g. more sensitive and responsive parenting) the greater the dyadic concordance in parent and offspring WM connectivity.

### Parental lifetime-MDD × early parental Care interaction

**Fig. 3.**
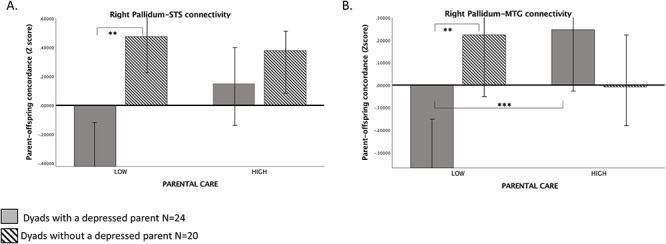
Graphical depiction of parental lifetime-MDD by parental Care interaction predicting dyadic concordance in parent and offspring WM pallidum-MTG and pallidum-STS connectivity. **P < 0.01; ***P < 0.001.N dyads (Low Care/Dyads with a depressed parent) = 13; N dyads (Low Care/Dyads without a depressed parent) = 11; N dyads (High Care/Dyads with a depressed parent) = 11; N dyads (High Care/Dyads without a depressed parent) = 9.

Finally, to test Hypothesis 4 whether high levels of early parental Care can protect the dyadic concordance from the effects of parental lifetime-MDD, we ran GEE analyses for the right Pallidum-MTG, right Pallidum-STS and right Pallidum-TPJ/SMG connections, the three CBG connections moderated by both parental lifetime-MDD and parental Care. To estimate the degree of concordance (similarity), we subtracted parents’ WM connectivity from the offspring’s WM connectivity and divided the absolute differences by the absolute sum of the two values (Törnqvist *et al.*, 1985). In each model, we entered parent lifetime-MDD status, parental Care and their interaction as predictors of the parent–offspring degree of concordance, and used familial (G1) risk status, offspring’s MDD status, parent’s and offspring’s age and sex as covariates. Significant parent lifetime-MDD × parental Care interactions were found predicting concordance in the right pallidum-STS connectivity (ß = − 0.45, SE = 0.19, *P* = 0.01) and in the right pallidum-MTG connectivity (ß = − 0.74, SE = 0.12, *P* < 0.0001). No significant interaction was found predicting concordance in the right Pallidum-TPJ/SMG connection (ß = − 0.34, SE = 0.25, *P* = 0.1). To illuminate the nature of significant interactions, we created bar charts of parental Care (high *vs* low) on parent–offspring concordance for dyads with a depressed parent and without a depressed parent and ran GEE regressions to examine associations between variables. As shown in Figure 3A, degree of right Pallidum-STS WM concordance was correlated with parental lifetime-MDD under low parental Care, (ß = 0.35, SE = 0.14, *P* < 0.01), indicating greater neural concordance among dyads with a non-depressed parent compared with dyads with a depressed parent, but no association was found under high parental Care (ß = 0.09, SE = 0.18, *P* > 0.05). No difference was found among dyads with and without a depressed parent in the degree WM concordance between low and high parental Care (ß = 0.31, SE = 0.25, *P* > 0.05; ß = − 0.002, SE = 0.10, *P* > 0.05, respectively). Dyads with a depressed parent and low parental Care had the weakest neural concordance. As shown in Figure 3B, degree of right Pallidum-MTG WM concordance was correlated with parental lifetime-MDD under low parental Care (ß = 0.48, SE = 0.14, *P* < 0.01), while no association was found under high parental Care (ß = 0.33, SE = 0.49, *P* > 0.05). Also, only among dyads with a depressed parent, there was a significant difference in the degree of WM concordance between low and high parental Care (ß = 0.49, SE = 0.18, *P* < 0.001), with a weaker concordance under low parental Care compared with high Care. No difference was found among dyads with a non-depressed parent (ß = − 0.13, SE = 0.41, *P* > 0.05). Again, dyads with a depressed parent and low parental Care had the weakest neural concordance.

Finally, in order to determine whether the current findings of the disruption in concordance among dyads with a depressed parent may be a risk factor for the intergenerational transmission of psychopathology and social impairment, or a consequence of parent and offspring depression, we conducted a second set of analyses, excluding all dyads with depressed offspring, and comparing two groups of dyads: non-depressed dyads (never-depressed parents and their never-depressed offspring) and at-risk dyads (depressed parents and their never-depressed offspring). Our findings were mainly consistent for all four hypotheses, suggesting disruption in concordance may be a potential mechanism underlying the intergenerational risk of depression. Details concerning the results of these analyses are presented in online supplementary material (see Supplementary Table S4,5).

## Discussion

One of the central pillars of human social development is the ability to be attuned to the social cues of others, including sharing others’ emotions and the ability to take into account others’ perspectives and thoughts, which is first experienced and formed in the context of early parent-child relationship and represented at physiological levels (Gordon *et al.*, 2020a, b; Decety, 2015). Such affective, behavioral and physiological attunements protect individuals from stressful experiences, unpredictable environment and from developing psychopathology from infancy to elderly (Barton et al., 2013; Stern *et al.*, 1985; Tops *et al.*, 2014; Feldman, 2020).

To our knowledge this is the first study to examine dyadic concordance in CBG circuits in a sample of mothers and fathers with and without a lifetime history of MDD and their offspring, and the first to show how neural concordance varies by risk factors—parental depression and poor early caregiving. We also examined if higher levels of parental Care, as perceived by the offspring during the first 16 years of life, provide a protective buffer against the attenuated concordance in WM connections associated with parental depression. Our study provides the first neurobiological evidence that parent–offspring concordance in specific WM connections between basal ganglia and temporal cortical areas implicated in mentalizing functions and which support social attachments (Meaney, 2001) is disrupted among families with a parental history of MDD, and its degree is associated with quality of early parental caregiving.

The basal ganglia structures are implicated in human attachments, and play a central role in the formation and maintenance of mother-offspring bonding, father-offspring bonding, co-parenting, romantic love and close friendship, due to the integration of OT and DA in these structures which ignites relationships, and imbuing attachments with motivation and rewarding (For review see: Feldman, 2015; Swain and Ho, 2017). However, the basal ganglia structures do not only maintain close interconnectivity, but are also connected via multiple ascending and descending projections to cortical regions, including the temporal cortex (Fareri and Delgado, 2013; Swain and Ho, 2017). Such connectivity allows the integration of later-evolving cortical structures implicated in higher order socio-affective processes with the conserved, automatic brain circuitry underpinning mammalian maternal care, motivation and novelty and reward seeking (Tops *et al.*, 2014; Feldman, 2015). It has been suggested that CBG circuits support human ability to appreciate social familiarity over novelty seeking, which is a vital process in promoting resilience and sociality and protecting against stress and psychopathology (Rilling and Young, 2014). For instance, in a recent fMRI longitudinal study, Abraham and colleagues (2016, 2017) found that connectivity between limbic network, including the basal ganglia structures, and cortical structures implicated in social cognition, when parents watched videos of their infants, shaped children’s OT response in preschool years, which in turn supported maturation of the child’s social competencies at school-entry. Also, parental CBG connectivity during infancy predicted lower child externalizing symptoms at 6 years as mediated by collaborative co-parenting in preschool years. Finally, Atzil and colleagues (2011) found that sensitive mothering was underpinned by greater connectivity between limbic structures and frontal and temporal regions.

In search for specific CBG pathways that define the integration of subcortical nuclei with cortical structures, our findings highlight three important aspects of human dyadic concordance and its links with psychopathology and social bonding. First, while using parental and offspring MDD status as covariates, we found concordance in associations between parent and offspring integrity of WM tracts that connect the basal ganglia nuclei with temporal regions (FG, ITG, TPJ, STS and TP). Second, we found that parental lifetime-MDD status and parental Care moderated these parent–offspring associations. Specifically, among dyads with a never-depressed parent, parents’ integrity of WM tracts that connect the basal ganglia nuclei with the temporal regions (STS, FG, MTG, TPJ and TP) were positively associated with those of their offspring. In contrast, among dyads with a depressed parent, no such associations were found. Also, higher levels of parental Care were associated with greater positive associations between parent and offspring integrity of WM tracks that connect the basal ganglia nuclei with FG, STS, MTG, ITG, TPJ and TP. Finally, parental Care was found to moderate the associations between parental lifetime-MDD and the degree of dyadic concordance in right pallidum-MTG and pallidum-STS. Under low levels of parental Care, greater concordance was found for dyads with a non-depressed parent compared with dyads with a depressed parent, while no differences between the two groups were found under high levels of parental Care. The weakest concordance was found among dyads with a depressed parent who also provided low parental Care, indicating that early parental caregiving may protect the dyadic concordance from the effects of parental lifetime-MDD. Also, for the right pallidum-MTG connection, among dyads with a depressed parent there was a significant difference in the degree of WM concordance between low and high parental Care, with a weaker concordance under low parental Care compared with high Care. No difference was found among dyads with a non-depressed parent.

Human attachment bonds have evolved on the basis of the early parent–offspring bonding (Hrdy, 2009), and are not merely bodily relations, but also brain-relations, ‘neural relations’. Human social bonding and affiliation throughout life are highly dependent on affect sharing, biobehavioral synchrony, self-other awareness, mental flexibility, perspective-taking, memory and language (Abraham *et al.*, 2014; Decety, 2015; de Waal and Preston, 2017; Feldman, 2017). Our finding of dyadic concordance in parent–offspring CBG WM connections and its link with early-life parent–offspring relationship adds to previous animal and human studies documenting the unique fit between biological and social processes that forms the specific attachment bond between parents and offspring (Ebisch *et al.*, 2012; Creaven *et al.*, 2014; Feldman *et al.*, 2010, 2011). In line with our results, recent neuroimaging studies reported stronger brain-to-brain coupling in basal ganglia and temporal cortex as reciprocity, familiarity and cooperation increased (Baker *et al.*, 2016; Reindl *et al.*, 2018), associations between neural coupling and affectionate bond (Atzil *et al.*, 2012; Levy *et al.*, 2017) and pain reduction (Goldstein *et al.*, 2018). For instance, a recent MEG study found that only during episodes of behavioral synchrony, compared with non-synchrony, mother’s and child’s STS gamma power was coupled (Levy *et al.*, 2017), two hyper-scanning fNIRS studies showed neural concordance in TPJ only during social gaze interaction, which was also related to the degree of dyadic reciprocity and cooperation (Mu *et al.*, 2016; Tang et al., 2016), and a hyper-scanning EEG study found a neural concordance among partners, but not strangers, localized in temporal-parietal structures, which was linked to behavioral synchrony among couples (Kinreich *et al.*, 2017). It has previously been shown that structural organizations follow the magnitude of neural activity, which means that tracts that are used more (function) will become denser and more organized (structure) and both are associated with behavioral performance (Bennett and Rypma, 2013; Burzynska *et al.*, 2013). Therefore, if structures are concordant, it stands to reason that there are also functional parallels, as found in previous studies examining coupling of neural activity. It will be necessary to test this assumption by demonstrating, for example, that concordant DTI measures correlate with a behavior measured in both the parent and the child.

We also found that parental depression disrupted parent–offspring WM concordance in CBG connections. This finding is in line with evidence showing that parental depression is associated with attenuated behavioral parent-child concordance (Field *et al.*, 1990; Murray and Cooper, 1996; Tronick and Reck, 2009; Granat *et al.*, 2017), which extends to the levels of hormonal (Merwin *et al.*, 2017), and autonomic (Woody *et al.*, 2016) functioning. It is worth noting that the connections between the basal ganglia and the temporal lobe, including the TPJ, TP and STS, are human-specific and stronger among humans compared with non-human primates, including chimpanzees, probably due to their role in social-interactive processes such as language, action observation and ToM (Innocenti *et al.*, 2016; de Waal and Preston, 2017). Indeed, parents suffering from depression tend to speak less, make less eye contact, generally slower in their responses to their children and exhibit lower positive affect and limited empathic understanding (Hummel *et al.*, 2016; Granat *et al.*, 2017).

Of significant interest is our finding that higher levels of early parental Care as, as reported by the offspring, buffered the attenuated neural concordance in the right pallidum-MTG and pallidum-STS associated with parental depression. This finding confirms the central role of the basal ganglia and the STS and MTG in the processing of attachment and social stimuli as well as their sensitivity to early environmental experiences (Brunet *et al.*, 2000; Cecchini *et al.*, 2013; Shields *et al.*, 2019). Also, finding is consistent with previous animal and human studies showing that social attachments, including parent–offspring bonding, pair-bonding and friendship, buffer against psychopathology and the consequences of stressful events, including exposure to parental depression (Flykt *et al.*, 2010; Pratt et al.,2015; Schury *et al.*, 2017; Vakrat *et al.*, 2018).

The pallidum comprises a rich neural circuitry of diverse cell types that shape both motor and non-motor features of behavior (Obeso *et al.*, 2008). In recent years, a plethora of studies has demonstrated that the pallidum is far more than a simple motor-control nucleus (Gittis *et al.*, 2014). Since the pallidum is rich in OT and DA receptors (Lim *et al.*, 2004; Avila *et al.*, 2020), it processes reward and motivational-related signals in general (Hong and Hikosaka, 2008), and specifically those related to social attachment (Bartels and Zeki, 2004; Leibenluft *et al.*, 2004). The pallidum supports maternal and paternal caregiving (Rilling and Mascaro, 2017), as well as children’s secure attachment to their parents (Choi *et al.*, 2018). The STS is a central region of the social cognition network, playing a vital role in mentalizing, parental care, biological motion, social goal interpretation, prediction making, and updating regarding others’ behavior (Gordon *et al.*, 2013; Abraham *et al.*, 2014; Wang *et al.*, 2018). The MTG, bounded by the STS above, is part of the ‘language network’, and together with the STS and subcortical structures, including the basal ganglia (Tomasi and Volkow, 2012) supports goal-directed action targeted at another person, and shows tight relationship with ToM functioning (Kandylaki *et al.*, 2015), with familiarity in the context of social attachment (Gobbini *et al.*, 2004) and with affective communication conveyed by empathic decoding of others’ pain experience (Lang *et al.*, 2011). Connectivity between the basal ganglia and MTG and STG have been shown to be associated with language processing (Booth *et al.*, 2007) and with attention to faces of social partners (Powell *et al.*, 2018)—two central components of human socio-affective communication and bonding (Hari and Kujala, 2009). Still, future studies should focus on the connections between the pallidum and the STS/MTG to obtain a better understanding of the functional and structural connectivity between these regions in social contexts in general, and in the context of early-life rearing experiences, in particular.

Finally, by excluding all dyads with depressed offspring and examining parent–offspring concordance between depressed and never-depressed parents and their never-depressed offspring in supplementary analyses, we suggest that such disruption in neural concordance within the CBG circuits may be a potential mechanism underlying the intergenerational risk of depression.

While this is the first study to examine dyadic concordance in CBG connections in a sample of parents with and without a history of MDD and their offspring, and the first to show how dyadic concordance in WM connectivity varies by parental risk factors using unique dyadic datasets with richly characterized clinical and environmental data over-time, several study limitations merit consideration. First, since our findings are based on a relatively small sample of parent–offspring dyads, future studies with a larger sample size are needed to further elucidate the nature of parental depression on neural concordance between parents and their offspring. Second, our findings are correlational in nature and do not suggest causality, and we are unable to test the directionality of our findings. Third, the sample was of European ancestry, as was the norm for family studies when the project originated; generalizability of the findings across racial and ethnic groups should not be assumed. Fourth, while the validity of the PBI has been supported by studies showing that subjects’ ratings correlate strongly with ratings of their parents themselves, siblings and impartial raters (Parker, 1984; Parker *et al.*, 1979; Steiger *et al.*, 1989), parental Care was based merely on offspring’s perception of relationship with their parents. Therefore, the interpretation of the results could be influenced by the fact that the instrument was self-reported and reflect a bias in the perception of parenting style. This could be particularly evident for our sample, in which the range of age of participants was broad. Future studies may consider integrating self-report and observational measures (e.g. coding parent–offspring social interaction) to evaluate parent–offspring bonding during early childhood.

To summarize, the present study provides important contributions to the extant literature by documenting parent–offspring concordance in WM structural connectivity across different developmental stages, which was found to be sensitive to parental psychopathology and early parental caregiving and provide another step in pinpointing the neurocircuitry that supports coordination between parent and offspring. Recent models in social neuroscience have proposed moving beyond studying neural processes at the within-individual level to considering coordinated neural activity across multiple individuals and how such neural coupling underpin the ability to form and maintain social bonds across the lifetime (Hari, 2017). Hopefully, our findings may stimulate more experimentally controlled studies to further examine dyadic concordance in other neurocircuitries, such as those implicated in fear, anxiety and aversive social processing. Future studies should explore how brain connectedness links with other psychiatric disorders, how other environmental experiences and disruptions of other social bonds interrupt brain coupling and should investigate other conditions under which brain synchronization operates to hinder or facilitate social bonding and functioning. Our findings may have important implications for advancing our understanding of disorders characterized by neural and behavioral disturbances in social cognition, such as major depression, as well as clinical implications for the development of early relationship‐focused interventions for depressed parents and their children to reduce the risk for psychopathology among at-risk populations.

## Supplementary Material

nsaa118_Supp

## References

[R1] Abraham E., Gilam G., Kanat-Maymon Y., et al (2017). The human coparental bond implicates distinct corticostriatal pathways: longitudinal impact on family formation and child well-being. *Neuropsychopharmacology*, 42(12), 2301. 10.1038/npp.2017.71 PMC5645748 28401924

[R2] Abraham E., Hendler T., Shapira-Lichter I., Kanat-Maymon Y., Zagoory-Sharon O., Feldman R. (2014). Father’s brain is sensitive to childcare experiences. *Proceedings of the National Academy of Sciences*, 111(27), 9792–97. 10.1073/pnas.1402569111 PMC4103311 24912146

[R3] Abraham E., Hendler T., Zagoory-Sharon O., Feldman R. (2016). Network integrity of the parental brain in infancy supports the development of children’s social competencies. *Social Cognitive and Affective Neuroscience*, 11(11), 1707–18. 27369068 10.1093/scan/nsw090 PMC5091682

[R4] Adolfi F., Couto B., Richter F., et al (2017). Convergence of interoception, emotion, and social cognition: A twofold fMRI meta-analysis and lesion approach. *Cortex*, 88, 124–42. 28088652 10.1016/j.cortex.2016.12.019

[R5] Alloy L.B., Abramson L.Y., Smith J.M., Gibb B.E., Neeren A.M. (2006). Role of parenting and maltreatment histories in unipolar and bipolar mood disorders: mediation by cognitive vulnerability to depression. *Clinical Child and Family Psychology Review*, 9(1), 23–64. 16718583 10.1007/s10567-006-0002-4

[R6] Andersson J.L.R., Sotiropoulos S.N. (2016). An integrated approach to correction for off-resonance effects and subject movement in diffusion MR imaging. *Neuroimage*, 125, 1063–78. 26481672 10.1016/j.neuroimage.2015.10.019 PMC4692656

[R7] Atzil S., Hendler T., Feldman R. (2011). Specifying the neurobiological basis of human attachment: brain, hormones, and behavior in synchronous and intrusive mothers. *Neuropsychopharmacology*, 36(13), 2603–15. 21881566 10.1038/npp.2011.172 PMC3230500

[R8] Atzil S., Hendler T., Zagoory-Sharon O., Winetraub Y., Feldman R. (2012). Synchrony and specificity in the maternal and the paternal brain: relations to oxytocin and vasopressin. *Journal of the American Academy of Child and Adolescent Psychiatry*, 51(8), 798–811. 22840551 10.1016/j.jaac.2012.06.008

[R9] Avila G., Picazo O., Chuc-Meza E., García-Ramirez M. (2020). Reduction of dopaminergic transmission in the globus pallidus increases anxiety-like behavior without altering motor activity. *Behavioural Brain Research*, 112589. 10.1016/j.bbr.2020.112589 32194191

[R10] Báez-Mendoza R., Schultz W. (2013). The role of the striatum in social behavior. *Frontiers in Neuroscience*, 7, 233. 10.3389/fnins.2013.00233 PMC3857563 24339801

[R11] Baker J.M., Liu N., Cui X., et al (2016). Sex differences in neural and behavioral signatures of cooperation revealed by fNIRS hyperscanning. *Scientific Reports*, 6, 26492. 10.1038/srep26492 PMC4897646 27270754

[RM0012] Bartels A., Zeki S. (2014). The neural correlates of maternal and romantic love. *Neuroimage*, 21(3), 1153–1166. 10.1016/j.neuroimage.2003.11.003 15006682

[R12] Barton Y.A., Miller L., Wickramaratne P., Gameroff M.J., Weissman M.M. (2013). Religious attendance and social adjustment as protective against depression: A 10-year prospective study. *Journal of Affective Disorders*, 146(1), 53–57. 22959684 10.1016/j.jad.2012.08.037 PMC3582716

[R13] Benjamini Y., Hochberg Y. (1995). Controlling the false discovery rate: a practical and powerful approach to multiple testing. *Journal of the Royal Statistical Society: Series B (Methodological)*, 57(1), 289–300.

[R14] Bennett I.J., Rypma B. (2013). Advances in functional neuroanatomy: a review of combined DTI and fMRI studies in healthy younger and older adults. *Neuroscience and Biobehavioral Reviews*, 37(7), 1201–10. 23628742 10.1016/j.neubiorev.2013.04.008 PMC3691337

[R15] Booth J.R., Wood L., Lu D., Houk J.C., Bitan T. (2007). The role of the basal ganglia and cerebellum in language processing. *Brain Research*, 1133, 136–44. 17189619 10.1016/j.brainres.2006.11.074 PMC2424405

[R16] Bowlby J. (1969). Attachment and loss v. 3 (Vol. 1). Random House. Furman, W., & Buhrmester, D. (2009). Methods and measures: the network of relationships inventory: behavioral systems version. *International Journal of Behavioral Development*, 33, 470–78. 10.1177/0165025409342634 PMC2826206 20186262

[R17] Brunet E., Sarfati Y., Hardy-Baylé M.-C., Decety J. (2000). A PET investigation of the attribution of intentions with a nonverbal task. *Neuroimage*, 11(2), 157–66. 10679187 10.1006/nimg.1999.0525

[R18] Burzynska A.Z., Garrett D.D., Preuschhof C., et al (2013). A scaffold for efficiency in the human brain. *The Journal of Neuroscience*, 33(43), 17150–59. 24155318 10.1523/JNEUROSCI.1426-13.2013 PMC6618437

[R19] Cecchini M., Aceto P., Altavilla D., Palumbo L., Lai C. (2013). The role of the eyes in processing an intact face and its scrambled image: A dense array ERP and low-resolution electromagnetic tomography (sLORETA) study. *Social Neuroscience*, 8(4), 314–25. 23706064 10.1080/17470919.2013.797020

[R20] Choi E.J., Taylor M.J., Hong S.-B., Kim C., Yi S.-H. (2018). The neural correlates of attachment security in typically developing children. *Brain and Cognition*, 124, 47–56. 29727768 10.1016/j.bandc.2018.04.003

[R21] Colich N.L., Ho T.C., Ellwood-Lowe M.E., et al (2017). Like mother like daughter: putamen activation as a mechanism underlying intergenerational risk for depression. *Social Cognitive and Affective Neuroscience*, 12(9), 1480–89. 28575505 10.1093/scan/nsx073 PMC5629825

[R22] Creaven A.-M., Skowron E.A., Hughes B.M., Howard S., Loken E. (2014). Dyadic concordance in mother and preschooler resting cardiovascular function varies by risk status. *Developmental Psychobiology*, 56(1), 142–52. 24022469 10.1002/dev.21098 PMC3963270

[R23] Cui X., Bryant D.M., Reiss A.L. (2012). NIRS-based hyperscanning reveals increased interpersonal coherence in superior frontal cortex during cooperation. *Neuroimage*, 59(3), 2430–37. 21933717 10.1016/j.neuroimage.2011.09.003 PMC3254802

[R24] Curley J.P., Champagne F.A. (2016). Influence of maternal care on the developing brain: mechanisms, temporal dynamics and sensitive periods. *Frontiers in Neuroendocrinology*, 40, 52–66. 26616341 10.1016/j.yfrne.2015.11.001 PMC4783284

[R25] de Waal F.B.M., Preston S.D. (2017). Mammalian empathy: behavioural manifestations and neural basis. *Nature Reviews Neuroscience*, 18(8), 498. 10.1038/nrn.2017.72 28655877

[R26] Decety J. (2015). The neural pathways, development and functions of empathy. *Current Opinion in Behavioral Sciences*, 3, 1–6.

[R27] Delgado M.R. (2007). Reward-Related Responses in the Human Striatum. *Annals of the New York Academy of Sciences*, 1104(1), 70–88. 17344522 10.1196/annals.1390.002

[R28] Dikker S., Silbert L.J., Hasson U., Zevin J.D. (2014). On the Same Wavelength: predictable Language Enhances Speaker-Listener Brain-to-Brain Synchrony in Posterior Superior Temporal Gyrus. *Journal of Neuroscience*, 34(18), 6267–72. 24790197 10.1523/JNEUROSCI.3796-13.2014 PMC4004812

[R29] Ebisch S.J., Aureli T., Bafunno D., Cardone D., Romani G.L., Merla A. (2012). Mother and child in synchrony: thermal facial imprints of autonomic contagion. *Biological Psychology*, 89(1), 123–9. 22001267 10.1016/j.biopsycho.2011.09.018

[R30] Fareri D.S., Delgado M.R. (2013). *Reward Learning: contributions of Corticobasal Ganglia Circuits to Reward Value Signals*. Cambridge University Press, New York: 444–64

[R32] Feldman R. (2012). Parent-infant synchrony: A biobehavioral model of mutual influences in the formation of affiliative bonds. *Monographs of the Society for Research in Child Development*, 77(2), 42–51.

[R33] Feldman R. (2015). The adaptive human parental brain: implications for children’s social development. *Trends in Neurosciences*, 38(6), 387–99. 25956962 10.1016/j.tins.2015.04.004

[R34] Feldman R. (2017). The neurobiology of human attachments. *Trends in Cognitive Sciences*, 21(2), 80–99. 28041836 10.1016/j.tics.2016.11.007

[R35] Feldman R. (2020). What is resilience: an affiliative neuroscience approach. *World Psychiatry*, 19(2), 132–50. 32394561 10.1002/wps.20729 PMC7215067

[R36] Feldman R., Gordon I., Zagoory-Sharon O. (2010). The cross-generation transmission of oxytocin in humans. *Hormones and Behavior*, 58(4), 669–76. 20558167 10.1016/j.yhbeh.2010.06.005

[R37] Feldman R., Magori-Cohen R., Galili G., Singer M., Louzoun Y. (2011). Mother and infant coordinate heart rhythms through episodes of interaction synchrony. *Infant Behavior & Development*, 34(4), 569–77. 21767879 10.1016/j.infbeh.2011.06.008

[R38] Field T., Healy B.T., Goldstein S., Guthertz M. (1990). Behavior-state matching and synchrony in mother-infant interactions of nondepressed versus depressed dyads. *Developmental Psychology*, 26(1), 7.

[R39] Flykt M., Kanninen K., Sinkkonen J., Punamäki R.L. (2010). Maternal depression and dyadic interaction: the role of maternal attachment style. *Infant and Child Development*, 19(5), 530–50.

[R40] Foland-Ross L.C., Behzadian N., LeMoult J., Gotlib I.H. (2016). Concordant patterns of brain structure in mothers with recurrent depression and their never-depressed daughters. *Developmental Neuroscience*, 38(2), 115–23. 27198667 10.1159/000444448 PMC4927380

[R41] Gabbay V., Ely B.A., Li Q., et al (2013). Striatum-based circuitry of adolescent depression and anhedonia. *Journalof the American Academy of Child and Adolescent Psychiatry*, 52(6), 628–41. 10.1016/j.jaac.2013.04.003 PMC3762469 23702452

[R42] Gittis A.H., Berke J.D., Bevan M.D., et al (2014). New roles for the external globus pallidus in basal ganglia circuits and behavior. *Journal of Neuroscience*, 34(46), 15178–83. 25392486 10.1523/JNEUROSCI.3252-14.2014 PMC4228126

[R43] Gobbini M.I., Leibenluft E., Santiago N., Haxby J.V. (2004). Social and emotional attachment in the neural representation of faces. *Neuroimage*, 22(4), 1628–35. 15275919 10.1016/j.neuroimage.2004.03.049

[R44] Goldstein P., Weissman-Fogel I., Dumas G., Shamay-Tsoory S.G. (2018). Brain-to-brain coupling during handholding is associated with pain reduction. *Proceedings of the National Academy of Sciences*, 115(11), E2528–37. 10.1073/pnas.1703643115 PMC5856497 29483250

[R45] Gordon I., Gilboa A., Cohen S., Kleinfeld T. (2020a). The relationship between physiological synchrony and motion energy synchrony during a joint group drumming task. *Physiology & Behavior*, 2020 Jul 11: 113074. 10.1016/j.physbeh.2020.113074 32663553

[R46] Gordon I., Vander Wyk B.C., Bennett R.H., et al (2013). Oxytocin enhances brain function in children withautism. *Proceedings of the National Academy of Sciences*, 110(52), 20953–8. 10.1073/pnas.1312857110 PMC3876263 24297883

[R47] Gordon I., Gilboa A., Cohen S., et al (2020b). Physiological and Behavioral Synchrony predict Group cohesion and performance. *Scientific Reports*, 10(1), 1–12. 32439861 10.1038/s41598-020-65670-1 PMC7242382

[R48] Granat A., Gadassi R., Gilboa-Schechtman E., Feldman R. (2017). Maternal depression and anxiety, social synchrony, and infant regulation of negative and positive emotions. *Emotion*, 17(1), 11. 10.1037/emo0000204 27441576

[R49] Hagan M.J., Roubinov D.S., Gress-Smith J., Luecken L.J., Sandler I.N., Wolchik S. (2011). Positive parenting during childhood moderates the impact of recent negative events on cortisol activity in parentally bereaved youth. *Psychopharmacology*, 214(1), 231–38. 20521029 10.1007/s00213-010-1889-5 PMC3562727

[R50] Hardy S. (2009). Mothers and others *The Evolutionary Origins of Mutual Understanding*. Cambridge, MA and London: Belknap Press of Harvard University Press.

[R51] Hasson U. (2004). Intersubject synchronization of cortical activity during natural vision. *Science*, 303(5664), 1634–40. 15016991 10.1126/science.1089506

[R52] Hari R. (2017). From brain–environment connections to temporal dynamics and social interaction: principles of human brain function. *Neuron*, 94(5), 1033–9. 28595047 10.1016/j.neuron.2017.04.007

[R53] Hari R., Kujala M.V. (2009). Brain basis of human social interaction: from concepts to brain imaging. *Physiological Reviews*, 89(2), 453–79. 19342612 10.1152/physrev.00041.2007

[RM0054] Hong S., Hikosaka O. (2008). The globus pallidus sends reward-related signals to the lateral habenula. *Neuron*, 60(4), 720–29. 19038227 10.1016/j.neuron.2008.09.035 PMC2638585

[R54] Hummel A.C., Kiel E.J., Zvirblyte S. (2016). Bidirectional effects of positive affect, warmth, and interactions between mothers with and without symptoms of depression and their toddlers. *Journal of Child and Family Studies*, 25(3), 781–9. 28757788 10.1007/s10826-015-0272-x PMC5527290

[R55] Innocenti G.M., Dyrby T.B., Andersen K.W., Rouiller E.M., Caminiti R. (2016). The crossed projection to the striatum in two species of monkey and in humans: behavioral and evolutionary significance. *Cerebral Cortex*, 27(6), 3217–30. 10.1093/cercor/bhw161 27282154

[R56] Jiang J., Dai B., Peng D., Zhu C., Liu L., Lu C. (2012). Neural synchronization during face-to-face communication. *Journal of Neuroscience*, 32(45), 16064–69. 23136442 10.1523/JNEUROSCI.2926-12.2012 PMC6621612

[R57] Kandylaki K.D., Nagels A., Tune S., Wiese R., Bornkessel-Schlesewsky I., Kircher T. (2015). Processing of false belief passages during natural story comprehension: an fMRI study. *Human Brain Mapping*, 36(11), 4231–46. 26356583 10.1002/hbm.22907 PMC6869249

[R58] Kaufman J., Birmaher B., Brent D., et al (1997). Schedule for affective disorders and schizophrenia for school-age children-present and lifetime version (K-SADS-PL): initial reliability and validity data. *Journal of the American Academy of Child and Adolescent Psychiatry*, 36(7), 980–8. 9204677 10.1097/00004583-199707000-00021

[R59] Kinreich S., Djalovski A., Kraus L., Louzoun Y., Feldman R. (2017). Brain-to-brain synchrony during naturalistic social interactions. *Scientific Reports*, 7(1), 17060. 10.1038/s41598-017-17339-5 PMC5719019 29213107

[R60] Lang S., Yu T., Markl A., Müller F., Kotchoubey B. (2011). Hearing others’ pain: neural activity related to empathy. *Cognitive, Affective & Behavioral Neuroscience*, 11(3), 386. 10.3758/s13415-011-0035-0 21533882

[R61] Leckman J.F. (1982). Best estimate of lifetime psychiatric diagnosis: A methodological study. *Archives of General Psychiatry*, 39(8), 879–83. 7103676 10.1001/archpsyc.1982.04290080001001

[RM0062] Leibenluft E., Gobbini M. I, Harrison T., Haxby J. V. (2004). Mothers’ neural activation in response to pictures of their children and other children. *Biological psychiatry*, 56(4), 225–32. 15312809 10.1016/j.biopsych.2004.05.017

[R62] Levy J., Goldstein A., Feldman R. (2017). Perception of social synchrony induces mother–child gamma coupling in the social brain. *Social Cognitive and Affective Neuroscience*, 12(7), 1036–46. 28402479 10.1093/scan/nsx032 PMC5490671

[R63] Liang K.-Y., Zeger S.L. (1986). Longitudinal data analysis using generalized linear models. *Biometrika*, 73(1), 13–22.

[R64] Lim M.M., Murphy A.Z., Young L.J. (2004). Ventral striatopallidal oxytocin and vasopressin V1a receptors in the monogamous prairie vole (Microtus ochrogaster). *Journal of Comparative Neurology*, 468(4), 555–70. 14689486 10.1002/cne.10973

[R65] Liu T., Saito G., Lin C., Saito H (2017). Inter-brain network underlying turn-based cooperation and competition: a hyperscanning study using near-infrared spectroscopy. *Scientific Reports*, 7(1), 1–12. 28819162 10.1038/s41598-017-09226-w PMC5561070

[R66] Long M., Verbeke W., Ein-Dor T., Vrtička P. (2020). A Functional Neuro-Anatomical Model of Human Attachment (NAMA): insights from First-and Second-Person Social Neuroscience. *Cortex*10.1016/j.cortex.2020.01.010 32092496

[R67] Ma C., Ding J., Li J., et al (2012). Resting-state functional connectivity bias of middle temporal gyrus and caudate with altered gray matter volume in major depression. *PLoS One*, 7, 9. 10.1371/journal.pone.0045263 PMC3454420 23028892

[R68] Mannuzza S., Fyer A.J., Klein D.F., Endicott J. (1986). Schedule for Affective Disorders and Schizophrenia—Lifetime Version modified for the study of anxiety disorders (SADS-LA): rationale and conceptual development. *Journal of Psychiatric Research*, 20(4), 317–25. 3806426 10.1016/0022-3956(86)90034-8

[R69] Marchand W.R., Lee J.N., Suchy Y., Johnson S., Thatcher J., Gale P. (2012). Aberrant functional connectivity of cortico-basal ganglia circuits in major depression. *Neuroscience Letters*, 514(1), 86–90. 22395089 10.1016/j.neulet.2012.02.063

[R70] Mark Cummings E., Keller P.S., Davies P.T. (2005). Towards a family process model of maternal and paternal depressive symptoms: exploring multiple relations with child and family functioning. *Journal of Child Psychology and Psychiatry*, 46(5), 479–89. 15845128 10.1111/j.1469-7610.2004.00368.x

[R71] Mayo O., Gordon I. (2020). In and out of synchrony—Behavioral and physiological dynamics of dyadic interpersonal coordination. *Psychophysiology*, 57(6), e13574. 10.1111/psyp.13574 32221984

[R72] McNeal N., Appleton K.M., Johnson A.K., et al (2017). The protective effects of social bonding on behavioral and pituitary-adrenal axis reactivity to chronic mild stress in prairie voles. *Stress*, 20(2), 175–82. 28276805 10.1080/10253890.2017.1295444 PMC5612411

[R73] Meaney M.J. (2001). Maternal care, gene expression, and the transmission of individual differences in stress reactivity across generations. *Annual Review of Neuroscience*, 24(1), 1161–92. 10.1146/annurev.neuro.24.1.1161 11520931

[R74] Merwin S.M., Smith V.C., Kushner M., Lemay E.P., Dougherty L.R. (2017). Parent-child adrenocortical concordance in early childhood: the moderating role of parental depression and child temperament. *Biological Psychology*, 124, 100–10. 28143803 10.1016/j.biopsycho.2017.01.013

[R75] Meyer M.L., Davachi L., Ochsner K.N., Lieberman M.D. (2019). Evidence that default network connectivity during rest consolidates social information. *Cerebral Cortex*, 29(5), 1910–20. 29668862 10.1093/cercor/bhy071

[R76] Miller J.G., Vrtička P., Cui X., et al (2019). Inter-brain synchrony in mother-child dyads during cooperation: an fNIRS hyperscanning study. *Neuropsychologia*, 124, 117–24. 30594570 10.1016/j.neuropsychologia.2018.12.021 PMC6937429

[R77] Mu Y., Guo C., Han S. (2016). Oxytocin enhances inter-brain synchrony during social coordination in male adults. *Social Cognitive and Affective Neuroscience*, 11(12), 1882–93. 27510498 10.1093/scan/nsw106 PMC5141958

[R78] Murphy E., Wickramaratne P., Weissman M. (2010). The stability of parental bonding reports: A 20-year follow-up. *Journal of Affective Disorders*, 125(1-3), 307–15. 20138671 10.1016/j.jad.2010.01.003 PMC2889015

[R79] Murray L., Cooper P.J. (1996). The impact of postpartum depression on child development. *International Review of Psychiatry*, 8(1), 55–63.

[R80] Nusslock R., Miller G.E. (2016). Early-life adversity and physical and emotional health across the lifespan: A neuroimmune network hypothesis. *Biological Psychiatry*, 80(1), 23–32. 26166230 10.1016/j.biopsych.2015.05.017 PMC4670279

[R81] Obeso J.A., Rodríguez-Oroz M.C., Benitez-Temino B., et al (2008). Functional organization of the basal ganglia: therapeutic implications for Parkinson’s disease. *Movement Disorders: Official Journal of the Movement Disorder Society*, 23(S3), S548-S559. 18781672 10.1002/mds.22062

[R82] Parker G. (1983).*Parental Overprotection: A Risk Factor in Psychosocial Development*. New York, Grune & Stratton.

[RM0082] Parker G. (1984). The measurement of pathogenic parental style and its relevance to psychiatric disorder. *Social Psychiatry*, 19(2), 75-81. 6719183 10.1007/BF00583818

[R83] Parker G. (1990). The Parental Bonding Instrument: A decade of research. *Social Psychiatry and Psychiatric Epidemiology: The International Journal for Research in Social and Genetic Epidemiology and Mental Health Services*, 25(1990), 281–2. 10.1007/BF00782881 2291129

[R84] Parker G., Tupling H., Brown L.B. (1979). A ParentalBonding Instrument. *British Journal of Medical Psychology*, 52, 1–10.

[R85] Perkeybile A.M., Bales K.L. (2017). Intergenerational transmission of sociality: the role of parents in shaping social behavior in monogamous and non-monogamous species. *Journal of Experimental Biology*, 220(1), 114–23. 28057834 10.1242/jeb.142182 PMC5278619

[R86] Powell L.J., Kosakowski H.L., Saxe R. (2018). Social origins of cortical face areas. *Trends in Cognitive Sciences*, 22(9), 752–63. 30041864 10.1016/j.tics.2018.06.009 PMC6098735

[R87] Pratt M., Apter-Levi Y., Vakart A., et al (2015). Maternal depression and child oxytocin response; moderation by maternal oxytocin and relational behavior. *Depression and Anxiety*, 32(9), 635–46. 26130435 10.1002/da.22392

[R88] Qu Y., Fuligni A.J., Galván A., Lieberman M.D., Telzer E.H. (2016). Links between parental depression and longitudinal changes in youths’ neural sensitivity to rewards. *Social Cognitive and Affective Neuroscience*, 11(8), 1262–71. 27013103 10.1093/scan/nsw035 PMC4967797

[R89] Reindl V., Gerloff C., Scharke W., Konrad K. (2018). Brain-to-brain synchrony in parent-child dyads and the relationship with emotion regulation revealed by fNIRS-based hyperscanning. *Neuroimage*, 178, 493–502. 29807152 10.1016/j.neuroimage.2018.05.060

[R90] Rilling J.K., Mascaro J.S. (2017). The neurobiology of fatherhood. *Current Opinion in Psychology*, 15, 26–32. 28813264 10.1016/j.copsyc.2017.02.013

[R91] Rilling J.K., Young L.J. (2014). The biology of mammalian parenting and its effect on offspring social development. *Science*, 345(6198), 771–76. 25124431 10.1126/science.1252723 PMC4306567

[R92] Schirmer A., Meck W.H., Penney T.B. (2016). The socio-temporal brain: connecting people in time. *Trends in Cognitive Sciences*, 20(10), 760–72. 27615804 10.1016/j.tics.2016.08.002

[R93] Schury K., Zimmermann J., Umlauft M., et al (2017). Childhood maltreatment, postnatal distress and the protective role of social support. *Child Abuse & Neglect*, 67, 228–39. 28282596 10.1016/j.chiabu.2017.02.021

[R94] Shamay-Tsoory S.G. (2011). The neural bases for empathy. *The Neuroscientist*, 17(1), 18–24. 21071616 10.1177/1073858410379268

[R95] Shields G.S., McCullough A.M., Ritchey M., Ranganath C., Yonelinas A.P. (2019). Stress and the medial temporal lobe at rest: functional connectivity is associated with both memory and cortisol. *Psychoneuroendocrinology*, 106, 138–46. 30981087 10.1016/j.psyneuen.2019.04.001 PMC6615559

[R96] Skelin I., Needham M.A., Molina L.M., Metz G.A.S., Gruber A.J. (2015). Multigenerational prenatal stress increases the coherence of brain signaling among cortico–striatal–limbic circuits in adult rats. *Neuroscience*, 289, 270–78. 25595989 10.1016/j.neuroscience.2015.01.009

[R97] Smith J.D., Woodhouse S.S., Clark C.A.C., Skowron E.A. (2016). Attachment status and mother–preschooler parasympathetic response to the strange situation procedure. *Biological Psychology*, 114, 39–48. 26738633 10.1016/j.biopsycho.2015.12.008 PMC4737994

[R98] Sroufe L. A. (1988). The role of infant-caregiver attachment in development. *In J. Belsky & T. Nezworski (Eds.), Clinical implications of attachment*, Hillsdale, NJ: Erlbaum, 18–38.

[R99] Suveg C., Braunstein West K., Davis M., Caughy M., Smith E.P., Oshri A. (2019). Symptoms and synchrony: mother and child internalizing problems moderate respiratory sinus arrhythmia concordance in mother–preadolescent dyads. *Developmental Psychology*, 55(2), 366. 10.1037/dev0000648 30507218

[R100] Stephens G.J., Silbert L.J., Hasson U. (2010). Speaker-listener neural coupling underlies successful communication. *Proceedings of the National Academy of Sciences*, 107(32), 14425–30. 10.1073/pnas.1008662107 PMC2922522 20660768

[R101] Steiger H., Feen J.V.D., Goldstein C., Leichner P. (1989). Defense styles and parental bonding in eating-disordered women. *International Journal of Eating Disorders*, 8(2), 131–40.

[R102] Stern D.N., Hofer L., Haft W., and Dore J. (1985). Affect attunement: the sharing of feeling states between mother and infant by means of inter-modal fluency. *In T. M. Field & N. A. Fox (Eds.), Social Perception in Infants*, Norwood, NJ: Ablex, 249–68.

[R103] Swain J.E., Ho S.-H.S. (2017). Neuroendocrine mechanisms for parental sensitivity: overview, recent advances and future directions. *Current Opinion in Psychology*, 15, 105–10. 28813249 10.1016/j.copsyc.2017.02.027 PMC7195810

[R104] Tang H., Mai X., Wang S., Zhu C., Krueger F., Liu C. (2016). Interpersonal brain synchronization in the right temporo-parietal junction during face-to-face economic exchange. *Social Cognitive and Affective Neuroscience*, 11(1), 23–32. 26211014 10.1093/scan/nsv092 PMC4692317

[R105] Tomasi D., Volkow N.D. (2012). Resting functional connectivity of language networks: characterization and reproducibility. *Molecular Psychiatry*, 17(8), 841–54. 22212597 10.1038/mp.2011.177 PMC3323720

[R106] Tops M., Koole S.L., IJzerman H., Buisman-Pijlman F.T.A. (2014). Why social attachment and oxytocin protect against addiction and stress: insights from the dynamics between ventral and dorsal corticostriatal systems. *Pharmacology, Biochemistry, and Behavior*, 119, 39–48. 10.1016/j.pbb.2013.07.015 23916423

[R107] Törnqvist L., Vartia P., Vartia Y.O. (1985). How should relative changes be measured? *The American Statistician*, 39(1), 43–6.

[R108] Tournier J.-D., Smith R., Raffelt D., et al (2019). MRtrix3: A fast, flexible and open software framework for medical image processing and visualisation. *Neuroimage*, 202, 116137. 10.1016/j.neuroimage.2019.116137 31473352

[R109] Tronick E., Reck C. (2009). Infants of depressed mothers. *Harvard Review of Psychiatry*, 17(2), 147–56. 19373622 10.1080/10673220902899714

[R110] Tse W.S., Bond A.J. (2004). The impact of depression on social skills: a review. *The Journal of Nervous and Mental Disease*, 192(4), 260–68. doi: 10.1097/01.nmd.0000120884.60002.2b 15060399

[R111] Tustison N.J., Avants B.B., Cook P.A., et al (2010). N4ITK: improved N3 bias correction. *IEEE Transactions on Medical Imaging*, 29, 1310–20. 20378467 10.1109/TMI.2010.2046908 PMC3071855

[R112] Ulmer-Yaniv A., Avitsur R., Kanat-Maymon Y., Schneiderman I., Zagoory-Sharon O., Feldman R. (2016). Affiliation, reward, and immune biomarkers coalesce to support social synchrony during periods of bond formation in humans. *Brain, Behavior, and Immunity*, 56, 130–39. 10.1016/j.bbi.2016.02.017 26902915

[R113] Vakrat A., Apter-Levy Y., Feldman R. (2018). Fathering moderates the effects of maternal depression on the family process. *Development and Psychopathology*, 30(1), 27–38. 28420452 10.1017/S095457941700044X

[R114] van Bakel H.J.A., Riksen-Walraven J.M. (2008). Adrenocortical and behavioral attunement in parents with 1-year-old infants. *Developmental Psychobiology: The Journal of theInternational Society for Developmental Psychobiology*, 50(2), 196–201. 10.1002/dev.20281 18286586

[R115] Van Den Bos W., Rodriguez C.A., Schweitzer J.B., McClure S.M. (2015). Adolescent impatience decreases with increased fron-tostriatal connectivity. *Proceedings of the National Academy of Sciences*, 112(29), E3765–74. 10.1073/pnas.1423095112 PMC4517266 26100897

[R116] van den Heuvel M.P., de Reus M.A., Feldman Barrett L., et al (2015). Comparison of diffusion tractography and tract-tracing measures of connectivity strength inrhesus macaque connectome. *Human Brain Mapping*, 36, 3064–75. 26058702 10.1002/hbm.22828 PMC6869766

[R117] Van Overwalle F. (2009). Social cognition and the brain: a meta‐analysis. *Human Brain Mapping*, 30(3), 829–58. 18381770 10.1002/hbm.20547 PMC6870808

[R118] Veraart J., Novikov D.S., Christiaens D., Ades-Aron B., Sijbers J., Fieremans E. (2016). Denoising of diffusionMRI using random matrix theory. *Neuroimage*, 142, 394–406. 27523449 10.1016/j.neuroimage.2016.08.016 PMC5159209

[R119] Wang Y., Metoki A., Alm K.H., Olson I.R. (2018). White matter pathways and social cognition. *Neuroscience and Biobehavioral Reviews*, 90, 350–70. 29684403 10.1016/j.neubiorev.2018.04.015 PMC5993647

[R120] Weissman M.M., Wickramaratne P., Gameroff M.J. et al (2016). Offspring of depressed parents: 30 years later. *American Journal of Psychiatry*, 173(10), 1024–32. 27113122 10.1176/appi.ajp.2016.15101327

[R121] Weissman M.M. (2020). Intergenerational study of depression: a convergence of findings and opportunities. *Psychological Medicine*, 50(1), 170–72. 31647040 10.1017/S0033291719002939

[R122] Wilhelm K.A.Y., Niven H., Parker G., Hadzi-Pavlovic D. (2005). The stability of the Parental Bonding Instrument over a 20-year period. *Psychological Medicine*, 35(3), 387–93. 15841874 10.1017/s0033291704003538

[R123] Woody M.L., Feurer C., Sosoo E.E., Hastings P.D., Gibb B.E. (2016). Synchrony of physiological activity during mother-child interaction: moderation by maternal history of major depressive disorder. *Journal of Child Psychology and Psychiatry*, 57(7), 843–50. 27090774 10.1111/jcpp.12562 PMC5680529

