# Supplementary material for: Concordance in parent and offspring cortico-basal ganglia white matter connectivity varies by parental history of major depressive disorder and early parental care
Source: Soc Cogn Affect Neurosci. 2020 Aug 25;15(8):889–903. doi: 10.1093/scan/nsaa118 (PMC7543940; doi:10.1093/scan/nsaa118)
Supplement: nsaa118_Supp [file nsaa118_supp.zip › nsaa118_Supp/scan-20-105-File003.docx]

**Supplementary Information**

Table S1. **Group differences in average rotation and average translations**

|  | High vs. low risk | Lifetime-MDD vs. No MDD | High vs. low parental care |
| --- | --- | --- | --- |
| **Parents** | | | |
| Avg_rot | t=1.235 (P=0.235) | t=0.706  (P=0.487) | t=1.484 (P=0.150) |
| Avg_trans | t=0.347 (P=0.732) | t=1.441  (P=0.162) | t=1.515 (P=0.142) |
| **Offspring** | | | |
| Avg_rot | t=0.304 (P=0.304) | t=-0.617  (P=0.541) | t=0.838  (P=0.408) |
| Avg_trans | t=0.611 (P=0.545) | t=-0.170  (P=0.866) | t=0.683  (P=0.499) |

Table S2 - **White matter connections in CBG circuits showing associations (concordance) between parents (with and without lifetime-MDD) and their offspring (with and without lifetime-MDD) and interaction effects between parent’s lifetime-MDD status x parent WM connectivity and between parental *Care* x parent WM connectivity in predicting offspring WM connectivity (All tested associations and interactions)**

| Basal ganglia region | CBG WM connections |  | L hemisphere | | | R  hemisphere | | |
| --- | --- | --- | --- | --- | --- | --- | --- | --- |
|  |  |  | Standardized ß (SE) | Z | P  value | Standardized ß (SE) | Z | P value |
| NAcc - | Fusiform gyrus | Parent WM connectivity | 0.38 (0.08) | 4.35 | <0.0001 | 0.08 (0.15) | 0.54 | 0.58 |
|  |  | 1. (a) Parental MDD   X  Parent WM connectivity | 0.39 (0.19) | 1.96 | 0.05 | 0.22 (0.16) | 1.39 | 0.16 |
|  |  | (b) Parental care  X  Parent WM connectivity | 0.45 (0.25) | -1.83 | 0.06 | -0.25 (0.19) | -1.29 | 0.29 |
|  | Interior temporal gyrus (ITG) | Parent WM connectivity | 0.34 (0.13) | 2.59 | 0.001 | 0.02 (1.84) | 0.12 | 0.9 |
|  |  | a | 0.25 (0.19) | 1.35 | 0.17 | 0.05 (0.19) | 0.27 | 0.78 |
|  |  | b | -0.09 (0.16) | -0.57 | 0.56 | -0.18 (0.17) | -1.07 | 0.28 |
|  | Middle temporal gyrus (MTG) | Parent WM connectivity | 0.16 (0.19) | 0.86 | 0.38 | 0.19 (0.13) | 1.49 | 0.13 |
|  |  | a | 0.42 (0.12) | 3.26 | 0.001 | 0.18 (0.11) | 1.52 | 0.12 |
|  |  | b | 0.11 (0.14) | 0.76 | 0.44 | -0.01 (0.16) | -0.12 | 0.90 |
|  | Temporal pole | Parent WM connectivity | -0.09 (0.15) | -0.59 | 0.5 | 0.48 (0.12) | 3.9 | <0.0001 |
|  |  | a | -0.50 (0.32) | -1.58 | 0.20 | -0.08 (0.12) | -0.67 | 0.50 |
|  |  | b | 0.68 (0.33) | 2.04 | 0.04 | -0.05 (0.19) | -0.28 | 0.77 |
|  | Superior temporal sulcus (STS) | Parent WM connectivity | 0.05 (0.17) | 0.32 | 0.74 | 0.18 (0.21) | 0.84 | 0.40 |
|  |  | a | 0.07 (0.24) | 0.30 | 0.76 | -0.29 (0.24) | -1.19 | 0.39 |
|  |  | b | 0.10 (0.24) | 0.42 | 0.67 | 1.57 (0.53) | 2.9 | 0.003 |
|  | TPJ/SMG | Parent WM connectivity | -0.09 (0.12) | -0.76 | 0.44 | -0.002 (0.12) | -0.02 | 0.98 |
|  |  | a | 0.009 (0.18) | 0.05 | 0.95 | 0.19 (0.17) | 1.12 | 0.26 |
|  |  | b | -0.002 (0.08) | -0.03 | 0.97 | -0.32 (0.31) | -1.01 | 0.31 |
| Caudate - | Fusiform gyrus | Parent WM connectivity | 0.39 (0.14) | 2.75 | 0.001 | 0.13 (0.13) | 1.01 | 0.31 |
|  |  | a | -0.04 (0.11) | -0.40 | 0.68 | 0.01 (0.12) | 0.14 | 0.88 |
|  |  | b | 0.45 (0.16) | 2.83 | 0.004 | -0.05 (0.10) | -0.34 | 0.73 |
|  | Interior temporal gyrus (ITG) | Parent WM connectivity | -0.006 (0.85) | -0.03 | 0.97 | -0.05 (0.14) | -0.39 | 0.69 |
|  |  | a | -0.18 (0.17) | -1.04 | 0.30 | -0.20 (0.21) | -0.95 | 0.34 |
|  |  | b | 0.10 (0.08) | 1.24 | 0.2 | -0.13 (0.15) | -0.86 | 0.40 |
|  | Middle temporal gyrus (MTG) | Parent WM connectivity | -0.14 (0.17) | -0.83 | 0.4 | 0.06 (0.11) | 0.56 | 0.57 |
|  |  | a | -0.02 (0.11) | 0.18 | 0.86 | 0.11 (0.20) | 0.56 | 0.57 |
|  |  | b | 0.45 (0.29) | 1.56 | 0.11 | 0.06 (0.15) | 0.42 | 0.67 |
|  | Temporal pole | Parent WM connectivity | -0.09 (0.13) | -0.73 | 0.46 | 0.07 (0.12) | 1.12 | 0.26 |
|  |  | a | -0.296 (0.19) | -1.55 | 0.12 | 1.06 (0.64) | 1.70 | 0.07 |
|  |  | b | 1.03 (0.32) | 3.17 | 0.001 | -0.08 (0.59) | -0.14 | 0.88 |
|  | Superior temporal sulcus (STS) | Parent WM connectivity | 0.24 (0.11) | 2.59 | 0.01 | -0.008 (0.11) | -0.08 | 0.93 |
|  |  | a | 0.04 (0.08) | 0.58 | 0.76 | -0.07 (0.17) | -0.46 | 0.64 |
|  |  | b | 0.10 (0.24) | 0.42 | 0.67 | 0.15 (0.13) | 1.14 | 0.26 |
|  | TPJ/SMG | Parent WM connectivity | 0.46 (0.25) | -0.76 | 0.06 | 0.03 (0.12) | 0.24 | 0.81 |
|  |  | a | 0.06 (0.30) | 0.21 | 0.83 | 0.15 (0.25) | 0.61 | 0.55 |
|  |  | b | 0.86 (0.44) | 1.93 | 0.005 | -0.02 (0.19) | -0.19 | 0.88 |
| Putamen - | Fusiform gyrus | Parent WM connectivity | 0.12 (0.24) | 0.52 | 0.6 | 0.28 (0.09) | 3.02 | 0.002 |
|  |  | a | 0.42 (0.12) | 3.48 | 0.0005 | 0.17 (0.27) | 0.66 | 0.51 |
|  |  | b | -0.02 (0.09) | -0.27 | 0.78 | 0.20 (0.19) | 1.06 | 0.29 |
|  | Interior temporal gyrus (ITG) | Parent WM connectivity | 0.23 (0.18) | 1.28 | 0.2 | 0.13 (0.86) | 0.71 | 0.47 |
|  |  | a | -0.01 (0.21) | -0.07 | 0.94 | -0.26 (0.34) | -1.28 | 0.40 |
|  |  | b | 0.33 (0.08) | 3.87 | 0.0001 | -0.27 (0.23) | -1.21 | 0.22 |
|  | Middle temporal gyrus (MTG) | Parent WM connectivity | 0.21 (0.37) | 0.57 | 0.56 | -0.08 (0.13) | -0.64 | 0.52 |
|  |  | a | 0.13 (0.10) | 1.28 | 0.20 | 0.20 (0.15) | 1.68 | 0.60 |
|  |  | b | 0.37 (0.23) | 1.61 | 0.10 | 0.13 (0.09) | 1.35 | 0.17 |
|  | Temporal pole | Parent WM connectivity | 0.08 (0.15) | 0.51 | 0.61 | 0.11 (0.18) | 0.63 | 0.53 |
|  |  | a | -0.05 (0.10) | -0.54 | 0.60 | 0.08 (0.17) | 0.51 | 0.6 |
|  |  | b | -0.20 (0.27) | -0.73 | 0.46 | -0.03 (0.12) | -0.26 | 0.8 |
|  | Superior temporal sulcus (STS) | Parent WM connectivity | 0.14 (0.14) | 1.0 | 0.31 | 0.06 (0.15) | 0.41 | 0.68 |
|  |  | a | 0.25 (0.12) | 2.07 | 0.03 | 0.05 (0.52) | 0.39 | 0.69 |
|  |  | b | 0.24 (0.15) | 1.60 | 0.10 | 0.23 (0.20) | 1.12 | 0.26 |
|  | TPJ/SMG | Parent WM connectivity | 0.22 (0.18) | 1.23 | 0.21 | 0.57 (0.09) | 6.16 | <0.0001 |
|  |  | a | -0.15 (0.14) | -1.02 | 0.40 | 0.25 (0.08) | 3.01 | 0.002 |
|  |  | b | 0.41 (0.08) | 4.93 | <0.0001 | -0.04 (0.12) | -0.36 | 0.71 |
| Pallidum - | Fusiform gyrus | Parent WM connectivity | 0.37 (0.16) | 2.30 | 0.02 | 0.10 (0.09) | 1.03 | 0.3 |
|  |  | a | 0.27 (0.15) | 1.83 | 0.06 | 0.04 (0.24) | 0.20 | 0.63 |
|  |  | b | 0.37 (0.32) | 1.16 | 0.24 | 1.42 (0.50) | 2.83 | 0.004 |
|  | Interior temporal gyrus (ITG) | Parent WM connectivity | -0.16 (0.13) | -1.20 | 0.39 | -0.03 (0.08) | -0.37 | 0.71 |
|  |  | a | 0.23 (0.24) | 0.98 | 0.32 | 0.55 (0.36) | 1.52 | 0.12 |
|  |  | b | -0.29 (0.28) | -1.03 | 0.8 | 0.61 (0.34) | 1.80 | 0.06 |
|  | Middle temporal gyrus (MTG) | Parent WM connectivity | 0.33 (0.56) | -0.58 | 0.56 | 0.26 (0.19) | 1.35 | 0.17 |
|  |  | a | 0.31 (0.38) | 0.81 | 0.41 | 0.30 (0.13) | 2.23 | 0.002 |
|  |  | b | 1.04 (0.80) | 1.29 | 0.19 | 0.52 (0.23) | 2.26 | 0.002 |
|  | Temporal pole | Parent WM connectivity | 0.31 (0.14) | 2.26 | 0.02 | 0.30 (0.07) | 4.18 | 0.0002 |
|  |  | a | -0.15 (0.18) | -0.86 | 0.38 | 0.05 (0.11) | 0.50 | 0.61 |
|  |  | b | 0.08 (0.10) | 0.83 | 0.40 | 0.38 (0.11) | 3.34 | 0.0008 |
|  | Superior temporal sulcus (STS) | Parent WM connectivity | 0.01 (0.18) | 0.10 | 0.91 | 0.28 (0.16) | 1.67 | 0.09 |
|  |  | a | 0.12 (0.17) | 0.73 | 0.46 | 0.33 (0.13) | 2.55 | 0.001 |
|  |  | b | 0.16 (0.12) | 1.31 | 0.19 | 0.58 (0.17) | 3.30 | 0.001 |
|  | TPJ/SMG | Parent WM connectivity | 0.49 (0.13) | 3.65 | 0.002 | 0.24 (0.09) | 2.56 | 0.01 |
|  |  | a | -0.13 (0.14) | -1.57 | 0.21 | 0.56 (0.26) | 2.12 | 0.003 |
|  |  | b | 0.52 (0.09) | 5.29 | <0.0001 | 0.85 (0.27) | 3.13 | 0.001 |

Note: significant associations and interactions after P<0.05 FDR correction (P<0.01); TPJ, temporoparietal junction; SMG, supramarginal gyrus.

Table S3- **Associations between parent-offspring concordance and parent age, sex, offspring MDD, sex and age**

| **Hemisphere** | **Cortical regions** | **Parent-offspring Concordance** | **Offspring MDD (0= MDD, 1= no-MDD)** | **Parent age** | **Offspring age** | **Offspring sex (0=male, 1=female)** | **Parent sex (0=male, 1=female)** |
| --- | --- | --- | --- | --- | --- | --- | --- |
| Left | STS | STS-Caudate (CA) | .093 | -.027 | .023 | .251 | .427^**^ |
|  |  | STS-Putamen (PU) | .103 | -.160 | -.243 | -.004 | -.208 |
|  |  | STS-Pallidum (PA) | .082 | -.018 | -.136 | .018 | -.144 |
|  |  | STS- NACC | .198 | -.012 | -.330^*^ | -.113 | .012 |
|  | FG | FG-CA | .344^*^ | .085 | .060 | -.007 | .342^*^ |
|  |  | FG-PU | .009 | .143 | .305^*^ | .091 | .284 |
|  |  | FG-PA | .231 | .126 | .073 | .093 | .090 |
|  |  | FG- NACC | -.085 | .025 | .137 | -.032 | .127 |
|  | ITG | ITG-CA | .141 | -.048 | .078 | .026 | .380^*^ |
|  |  | ITG-PU | .129 | .197 | .123 | .164 | .102 |
|  |  | ITG-PA | .143 | .052 | -.022 | .147 | -.002 |
|  |  | ITG- NACC | .020 | -.040 | .058 | .003 | .031 |
|  | MTG | MTG-CA | .023 | -.127 | -.005 | .042 | .442^**^ |
|  |  | MTG-PU | .319^*^ | .199 | .072 | .101 | .035 |
|  |  | MTG-PA | .215 | .151 | .076 | .037 | .023 |
|  |  | MTG- NACC | -.099 | -.161 | -.012 | .015 | .236 |
|  | Temporal pole (TP) | TP-CA | .197 | .204 | .327^*^ | .202 | .032 |
|  |  | TP-PU | -.010 | .076 | .141 | .160 | .126 |
|  |  | TP-PA | .207 | -.192 | -.192 | -.147 | -.153 |
|  |  | TP- NACC | -.307^*^ | .107 | .190 | .015 | .047 |
|  | TPJ/SMG | TPJ-CA | .218 | -.019 | -.057 | .155 | .370^*^ |
|  |  | TPJ-PU | .245 | .317^*^ | .048 | .337^*^ | .365^*^ |
|  |  | TPJ-PA | .237 | .093 | -.144 | .167 | .373^*^ |
|  |  | TPJ- NACC | -.114 | -.251 | -.233 | -.035 | .134 |
| Right | STS | STS-CA | .064 | -.057 | .046 | -.199 | .338^*^ |
|  |  | STS-PU | .194 | .114 | -.127 | .062 | .094 |
|  |  | STS-PA | .183 | .051 | -.176 | .010 | -.022 |
|  |  | STS- NACC | .147 | .188 | .152 | -.072 | .028 |
|  | FG | FG-CA | .046 | .039 | -.212 | .304^*^ | -.131 |
|  |  | FG-PU | .403^**^ | .029 | .139 | .185 | .289 |
|  |  | FG-PA | .045 | .191 | .094 | .279 | -.313^*^ |
|  |  | FG- NACC | -.125 | .180 | .210 | .171 | .111 |
|  | ITG | ITG-CA | .229 | .095 | -.071 | .257 | .328^*^ |
|  |  | ITG-PU | .324^*^ | .066 | .046 | .170 | .205 |
|  |  | ITG-PA | .225 | .254 | -.022 | -.069 | -.095 |
|  |  | ITG- NACC | -.010 | .135 | -.126 | .140 | -.290 |
|  | MTG | MTG-CA | .262 | -.016 | -.062 | -.125 | .542^***^ |
|  |  | MTG-PA | .152 | -.149 | .000 | .030 | -.258 |
|  |  | MTG- NACC | -.136 | -.060 | -.035 | -.009 | .334^*^ |
|  |  | MTG-PU | -.044 | .058 | .052 | .277 | .125 |
|  | TPJ/SMG | TPJ-CA | .218 | -.019 | -.057 | .155 | .370^*^ |
|  |  | TPJ-PA | .237 | -.093 | -.144 | .167 | .373^*^ |
|  |  | TPJ-PU | .245 | .317^*^ | .048 | -.337^*^ | .365^*^ |
|  |  | TPJ- NACC | -.114 | -.251 | -.233 | -.035 | .134 |
|  | TP | TP-NACC | -.143 | .181 | .013 | .258 | .073 |
|  |  | TP-PU | -.002 | .168 | .106 | -.051 | -.102 |
|  |  | TP-PA | .037 | -.029 | -.110 | .072 | -.226 |
|  |  | TP-CA | .115 | .209 | .014 | .205 | .130 |

Note: *P<0.05; **P<0.01; *** P<0.001

Table S4. **White matter connections in CBG circuits showing significant positive associations**† **(concordance) between parents (with and without lifetime-MDD) and their never-MDD offspring**

| Cortical Regions of Interest | CBG WM connections | L hemisphere |  | R hemisphere |  |
| --- | --- | --- | --- | --- | --- |
|  |  | Standardized ß (SE) | Z | Standardized ß (SE) | Z |
| STS | STS-Putamen | 0.451 (0.135) | 3.33*** | ns | ns |
| MTG |  | ns | ns | ns | ns |
| FG/ITG |  | ns | ns | ns | ns |
| TPJ/SMG | TPJ-Putamen | 0.472 (0.119) | 3.95**** | 0.642 (0.135) | 4.73**** |
|  | TPJ-Pallidum | 0.596 (0.157) | 3.79**** | ns | ns |

ns=non-significant after Bonferroni-correction; *** P<0.001; **** P<0.0001; Significant after P<0.05 FDR correction. Controlled for: lifetime-MDD status, offspring sex and age. STS, superior temporal sulcus; FG, fusiform gyrus; STS, superior temporal sulcus; MTG middle temporal gyrus; ITG, inferior temporal gyrus; TPJ, temporoparietal junction; SMG, supramarginal gyrus

Table S5. **White matter connections in CBG circuits showing: A. Significant† interaction effects between parent’s lifetime-MDD status x parent WM connectivity and**

**B. Significant† interaction effects between parental care x parent WM connectivity in predicting offspring WM connectivity (only non-depressed offspring)**

| CBG WM connections | parent lifetime-MDD status  x  parent WM connectivity  Standardized ß (SE) | Z |
| --- | --- | --- |
| L hemisphere | | |
| NAcc-MTG | 0.547 (0.094) | 5.83**** |
| Pallidum-FG/ITG | 0.621 (0.134) | 4.63**** |
| R hemisphere | | |
| **Temporo-Parietal cortex** |  |  |
| Pallidum-TPJ/SMG | 1.626 (0.226) | 7.17**** |
| **Pallidum-MTG** | **0.665 (0.135)** | **4.93****** |
| **Pallidum-FG/ITG** | **0.559 (0.098)** | **5.67****** |
| Putamen-TPJ/SMG | 0.610 (0.054) | 11.4**** |
| CBG WM connections | Parental care  x  parent WM connectivity  Standardized ß (SE)  (Outcome: offspring WM connectivity) | Z |
| L hemisphere | | |
| Putamen-FG/ITG | 0.404 (0.105) | 3.85**** |
| Pallidum-TPJ/SMG | 0.362 (0.129) | 2.79**** |
| R hemisphere | | |
| **Pallidum-MTG** | **0.817 (0.242)** | **3.37****** |
| **Pallidum-FG/ITG** | **0.738 (0.114)** | **6.43****** |
| Pallidum-TPJ/SMG | 0.907 (0.340) | 2.66*** |

*** P<0.001; ****P<0.0001; **Table shows only significant interactions after FDR correction P<0.05.** Controlled for: parent lifetime-MDD, offspring sex and age. Parental MDD status: MDD=1. ^#^ CBG connections moderated by both parental lifetime-MDD and parental care. STS, superior temporal sulcus; FG, fusiform gyrus; STS, superior temporal sulcus; MTG middle temporal gyrus; ITG, inferior temporal gyrus; TPJ, temporoparietal junction; SMG, supramarginal gyrus
